# Supplementary material for: Chromothripsis during telomere crisis is independent of NHEJ, and consistent with a replicative origin
Source: Genome Res. 2019 May;29(5):737–49. doi: 10.1101/gr.240705.118 (PMC6499312; doi:10.1101/gr.240705.118)
Supplement: Supplemental Material [file supp_gr.240705.118_Supplemental_file_1.zip › contigs/annotated_contigs/DB55/contig.2.DB55_length_293_mean_cov_25.9180887372.docx]

**DB55_length_293_mean_cov_25.9180887372**

```
TGCCGTCTCAGCTTCCCAAGTAGCTGGGACTACAGGCATGTGCCAAAAAA|TT|TTGAGCGCCGACTGTGTGCAGGGATTGGTTGAGCG
 >chrX:2756026-2756076 + E=1e-18                     >chr16:1142913-1143137 - E=4e-94
CCGACTGTGTGCAGGGATTGGTTGAGCGCCGACTGTGTGCAGTGATTGGTTGAGCGCCGACTGTGTGCTGTGATTGGTTGAGCGCTGTG

TGCACGGATTGGTTGAGCGCCGACTGTGTGCAGGGATTGGTTGAGCGCCGACTGTGTGCTGTGATTGGTTGAGCGCCGACTGTGTGCAG

TGATTTG|CATCTGAAATAAATCAAATAT
```
